# The Effects of Exercising in Different Natural Environments on Psycho-Physiological Outcomes in Post-Menopausal Women: A Simulation Study

**DOI:** 10.3390/ijerph120911929

**Published:** 2015-09-23

**Authors:** Mathew P. White, Sabine Pahl, Katherine J. Ashbullby, Francesca Burton, Michael H. Depledge

**Affiliations:** 1 European Centre for Environment and Human Health, University of Exeter Medical School, Knowledge Spa, Royal Cornwall Hospital, Truro, TR1 3 HD, UK; E-Mails: katherine.ashbullby@pcmd.ac.uk (K.J.A.); M.depledge@exeter.ac.uk (M.H.D.); 2 Department of Psychology, Plymouth University, Plymouth, PL4 8AA, UK; E-Mails: sabine.pahl@plymouth.ac.uk (S.P.); F.pimm@hotmail.co.uk (F.B.)

**Keywords:** physical activity, natural environments, green exercise, blue exercise

## Abstract

The current study examined potential psycho-physiological benefits from exercising in simulated natural environments among a sample of post-menopausal women using a laboratory based protocol. Participants cycled on a stationary exercise bike for 15 min while facing either a blank wall (Control) or while watching one of three videos: Urban (Grey), Countryside (Green), Coast (Blue). Blood pressure, heart rate and affective responses were measured pre-post. Heart rate, affect, perceived exertion and time perception were also measured at 5, 10 and 15 min during exercise. Experience evaluation was measured at the end. Replicating most earlier findings, affective, but not physiological, outcomes were more positive for exercise in the simulated Green and, for the first time, Blue environment, compared to Control. Moreover, only the simulated Blue environment was associated with shorter perceived exercise duration than Control and participants were most willing to repeat exercise in the Blue setting. The current research extended earlier work by exploring the effects of “blue exercise” and by using a demographic with relatively low average levels of physical activity. That this sample of postmenopausal women were most willing to repeat a bout of exercise in a simulated Blue environment may be important for physical activity promotion in this cohort.

## 1. Introduction

Moderate physical activity, including walking or cycling, offers a range of benefits for health and well-being [1,2]. However, until recently, where the physical activity took place received little attention. Although ecological models of health behavior consider characteristics of the physical environment, these tend to be defined very broadly, e.g., in terms of place of residence and accessibility of dedicated sports facilities such as swimming pools [3]. However, research is lacking that compares the effect of exercise taking place in different settings. Simultaneously, there has been increasing recognition in the field of Environmental Psychology that exposure to natural environments such as parks and woodlands (“green environments”) and rivers and coastlines (“blue environments”) benefits health and well-being [4,5,6,7]. Given these two strands of evidence, efforts have begun to explore the potential benefits of undertaking physical activity in various settings. Put simply, might physical activity in natural settings be more beneficial than similar exercise in indoor gyms or urban settings? This question relates keenly to a recent paradigm shift that emphasises the role of affect in exercise behavior as opposed to cognitive factors [8,9]. While these researchers focus on the affect arising directly from the experience of physical exercise, the present paper extends this reasoning by investigating whether positive affect from exercise varies by the setting of the exercise. 

### 1.1. Evidence of Interactive Effects

In a pioneering study, Pretty and colleagues [10] investigated the interactive effects of viewing different (simulated) environments (pleasant and unpleasant, urban and natural, scenes) while engaging in physical activity in a laboratory setting, on a range of physiological and psychological outcomes. Results suggested that, compared to a control condition (a blank wall), viewing a pleasant rural scene while exercising on a treadmill led to a significant decrease in mean arterial blood pressure, whereas viewing an unpleasant urban scene led to a significant increase. Moreover, self-esteem was significantly higher following exercise while viewing pleasant rural and urban scenes than control exercise, suggesting that both physiological and psychological outcomes may be affected by the setting in which exercise takes place. 

Since 2005 a sufficient body of work has been conducted to result in two systematic reviews of the potential interactive effects of physical activity across different environments. Thompson Coon *et al.* [11] compared the impacts on physical and mental wellbeing of physical activity indoors, mostly on treadmills, with physical activity undertaken in outdoor natural settings. A second review by Bowler *et al.* [12] focused more broadly on natural *versus* non-natural settings, including indoor and outdoor urban environments, and active and passive exposure. Both reviews concluded that few studies in this area were well conducted, but they nonetheless came to broadly the same two conclusions. 

First, they agreed that there was support for the notion that exposure to, and especially practice of physical activity in, natural environments leads to better affective outcomes, including increased feelings of revitalization, positive engagement and energy, and decreased negative affect, tension, confusion, anger and depression. This is consistent with earlier suggestions that natural environments promote stress reduction [13,14,15,16]. Second, there was little prior evidence of interactive effects of nature and physical activity on either physiological outcomes (e.g., heart rate or blood pressure) or cognitive processes (e.g., attention tasks).

Importantly, the reviews identified a number of gaps in the literature that require further research. For instance, most studies were conducted with convenience samples, primarily: “*college students, adult males, and physically active adults, and are not representative*” ([12], p. 7). Levels of physical activity and cardiovascular fitness tend to be highest in this age group [17] and consequently it may be difficult to detect environmental effects on physiological outcomes. There is also evidence that connectedness with natural environments is lowest during adolescence and early adulthood [18,19] and therefore any effects of different environments might be weaker and less evident among this demographic. 

Further, both reviews had difficulty identifying exactly what environments were being compared, both within and across studies: “*There is a limited variety of types of outdoor space utilized for physical activity and little descriptive information on the outdoor space provided in most papers”* ([11], p. 1764). Most studies were on “university campuses”, which presumably contain a mixture of buildings, roads and green space, while others were simply described as being “outdoors”. These limited descriptions, added to a small number of environment types used as comparators, make it difficult to construct a picture of the relative impacts of specific environments.

While examining physical activity across different environments, most studies also only monitored outcomes pre and post exercise (although see [20,21]). This is unfortunate as key changes may occur during the exercise period itself, but dissipate by the time of post-testing. Building on the circumplex model of affect [22], which argues that affective states fall along the perimeter of a circle with two dimensions characterized by valence (positive-negative) and arousal/activation, Ekkekakis and colleagues [23] developed a method for monitoring affective responses in terms of both valence and arousal at several times before, during and after exercise. Using this approach, Focht [20] investigated changes in affect before, during and after a 10 min walk, either in the laboratory or “outdoors”, among a cohort of young female volunteers. Although both walks resulted in improvements in positive affect during and after the walks, the outdoor walk had a larger impact *during* the walk. 

Focht [20] also asked participants how much they enjoyed the walk and their willingness to walk in “similar settings” in the future. These evaluative and intention related items are important in understanding how affective experiences during physical activity might translate into future behaviour, especially intentions to engage in further physical activity. As predicted, “outdoor” walking resulted in more positive evaluations and intentions to walk in similar settings again. However, as “outdoor” was defined as “a standardized route on sidewalks and walking paths in the area immediately surrounding the building” (p. 614) it remains unclear whether all kinds of outdoor environment have the same impact.

Although not explicitly mentioned in either review, none of the studies considered time perception during bouts of exercise in the different environments. The idea that people can experience a sense of flow [24] during exercise is not new [25]. What is novel is the possibility that it is easier to experience flow and lose track of time while exercising in a natural settings than in an indoor “Gym”. If natural settings are intrinsically fascinating and pleasurable [26], and time passes more quickly when experiencing positive affective states [27], then it should be easier for the mind to lose track of time in natural environments. A similar process might also apply for perceptions of effort, as measured by the Rate of Perceived Exertion scale (RPE, [28,29]). If people become immersed in an environment, not only might they lose track of time but they might also lose track of effort. This could be crucial because if people are likely to become more immersed in some environments than others, they may continue exercising for longer and harder without realizing it, with resulting benefits for health.

### 1.2. Current Research

Our aim was to explore the potential interactive effects between physical activity and various natural and non-natural settings by: (a) using a non-student sample of post-menopausal women; (b) using a laboratory study design to compare a range of simulated “outdoor” Urban (Grey), Countryside (Green) and Coastal (Blue) environments with a neutral indoor “Control” environment; (c) monitoring physiological and psychological outcomes before, during and after, exercise; and (d) examining whether physical activity in different simulated environments is associated with different time and physical effort perceptions.

Post-menopausal women are an important demographic for this research because compared to the usual samples of students they are: (a) older and possibly more sensitive to environmental contexts [30]; (b) tend to engage in less physical activity [31] and therefore may show more physiological heterogeneity in their responses to physical activity in different environments; and (c) because they tend to engage in less physical activity, they are a key target group for physical activity promotion interventions [32]. Identifying the optimal environment for physical activity among this demographic could facilitate more targeted health promotion programmes. Finally, a previous study, using a similar cohort, found that affect (as measured by the Positive and Negative Affect Scale) improved more following a 60 min walk “outdoors” than on an indoor treadmill, demonstrating the importance of context for physical activity for this demographic [33].

In addition to exploring the role of (simulated) natural environments on exercise outcomes by using a countryside (Green) setting, we also included a coastal (Blue) setting. Aquatic (blue) spaces are associated with a range of positive outcomes, over and above green spaces [34,35,36]. Moreover, people who live near the coast are more likely to engage in physical exercise than those living inland [3,37,38]. This may explain why coastal inhabitants have higher overall levels of self-reported health and well-being ([7,39], although see [40]). Consequently, if the opportunity is available to undertake physical activity near water it may be particularly beneficial. By also examining the potential interactive effects of physical activity and Blue environments, we again extend investigations in this area. Including a simulated urban (Grey) environment in the study design enabled us to examine whether any video of an outdoors environment would be more beneficial than a neutral indoor Control setting.

Following Teas *et al.* [33], a repeated measures, cross-over experimental design was used, involving a sample of post-menopausal women. Following Pretty *et al.* [10], the role of natural environments during physical activity was investigated in a laboratory setting using projections of different environments (in this case videos) onto the wall in front of participants while they engaged in exercise on stationary equipment. An obvious disadvantage with simulated environments is reduced ecological validity [41] although laboratory approaches are widely used in exercise research [32]. Although Bowler *et al.* [12] only reviewed studies involving direct exposure to natural environments, they nonetheless acknowledged that controlled laboratory simulations are useful for reducing biases that may be present in field testing, and Kerr and colleagues [42] found the difference in responses to physical activity between laboratory and outdoor conditions to be minimal (see also [43]). 

Stationary cycling was used as the form of physical activity in the study for several reasons. First, it was a lower impact form of exercise for our cohort of middle to older aged women, compared to say jogging. Second, in their study of green exercise *in situ*, Mackay and Neill [44] found that road and mountain biking were better at reducing anxiety compared to outdoor pursuits involving running or jogging. Although the authors offer no explanations for these results, it may be that the opportunity to free wheel and lower impact on joints may play a role. Finally, by using a stationary bike in a laboratory setting, rather than on roads outside, a much greater level of control could be applied to the study, for instance there were no traffic, gradient or weather issues (see also [45]). This not only ensured that all participants experienced identical environmental stimuli, it was also much physically safer (something the ethics committee were concerned about with our cohort). 

### 1.3. Hypotheses and Research Questions

Our hypotheses focused on comparing the outcomes of physical activity undertaken in the two simulated natural environment settings (“Green” and “Blue”) with the neutral “Control” environment to examine whether there were any additional benefits of exercising in these settings over simply the exercise *per se*. The simulated urban “Grey” environment was used as an additional control setting to see whether any distraction, even a busy urban one, during physical activity could have positive effects. The Control-Urban comparisons were thus more exploratory in nature. 

Specifically, and building directly on Pretty *et al.* [10], we predicted that compared to physical activity in the neutral Control condition, activity in the simulated natural setting conditions (Green and Blue) would be associated with: (H1) more positive physiological outcomes (*i.e.*, greater pre-post drops in Mean Arterial Blood Pressure and Heart Rate) after exercise; (H2) better affective responses (*i.e.*, more positive valence and lower arousal) both during and after exercise; (H3) shorter perceptions of time and lower perceptions of effort during exercise; and (H4) more positive global evaluations (*i.e.*, greater enjoyment and willingness to repeat) post exercise. Further, also following Pretty *et al.* [10], we predicted that: (H5) the Urban (Grey) condition may result in worse physiological and psychological outcomes than the Control condition and thus support the contention that any benefits from watching natural scenes are not merely due to the distraction effects of viewing any simulated environment while engaging in stationary exercise, but are instead associated with the specific environments encountered. 

## 2. Method 

### 2.1. Participants 

Thirty-seven post-menopausal women (Mean age = 50.11, SD = 3.69) participated in the study. Mean BMI was 25.33 (SD = 4.70). The mean number of self-reported instances of 30 min or more episodes of light to moderate exercise per week was 4.48 (SD = 2.93). All participants were retained throughout the study, a 0% drop-out rate. 

### 2.2. Design 

A repeated measures experimental design was employed. Each participant participated in four trials (Control, Urban, Green and Blue environments) with order randomised. Trials consisted of cycling for 15 min on a fixed exercise bike in an indoor laboratory at a comfortable pace. Trials took place approximately a week apart. Ethical approval was granted by the University of Exeter Medical School. 

### 2.3. Procedure and Materials

#### 2.3.1. Recruitment and Familiarization

Participants were recruited via posters, local newspapers and social media. Potential participants phoned the team and arranged a date for a familiarization session. This session took place a week before the first trial. During familiarization, the research process was explained and participants were introduced to all the measures they would subsequently be asked to respond to. If they were satisfied with what would be expected of them, they were asked to sign an informed consent form. A health screening questionnaire was completed to ensure no participants had contra-indications to exercise. Participant’s height, weight and resting heart-rate were measured and the correct height and handlebar setting on the bike was recorded for each individual (based on hip to saddle height and stretch to handlebars). To prepare for the trials, participants were asked to sit on the bike and cycle at a “light to moderate intensity” such that “you should feel comfortable holding a conversation during the ride, but at the end of the 15 min you may be slightly out of breath and slightly sweaty.” To ensure participants continued cycling at a light to moderate intensity, a revolutions per minute (RPM) range was derived for each participant based on their beginning the exercise at 55%–60% of their maximum heart rate, adjusting for age. If necessary, during trials the experimenter encouraged participants to speed up or slow down to stay within the RPM range. Water, a fan and a towel were available in the familiarization and trial sessions if participants wanted them. 

#### 2.3.2. Equipment and Materials 

**Figure 1:**
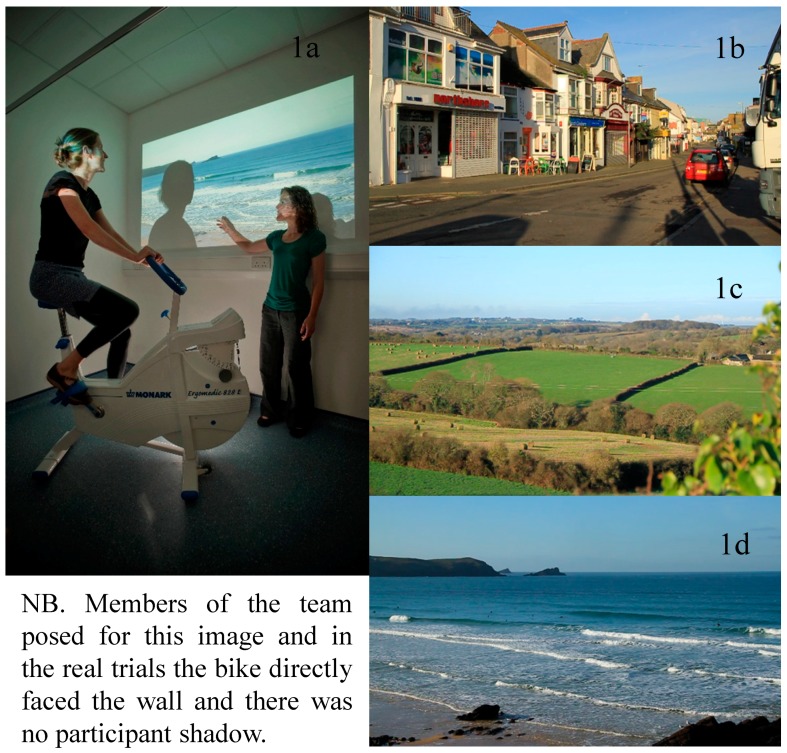
An image of the laboratory where the study took place (**1a**) and screenshots from the three “outdoor” videos (**1b**–**d**).

The bike was a Monark ergomedic ergometer stationary bike (828E). In the neutral Control condition participants faced a blank white wall, approximately 5 feet in front of them and taking up nearly their entire field of vision. In the experimental conditions one of three videos was projected onto the same wall (approx. 7 feet wide by 5 feet high). The videos consisted of 15 min of footage from Urban, Green or Blue settings. All footage was recorded by a professional videographer using a Canon 7D digital SLR in HD movie mode (Figure 1). Audio was recorded with a Zoom H1 external stereo microphone. Footage was taken on a mounted tripod set at approximately eye-level. There was no panning and the views were fixed (*i.e.*, not moving forward to simulate a cycle ride). All videos were filmed in Southwest England in January 2012 on sunny days to ensure climatic conditions were comparable.The Urban video included three 5 min clips of streets/pedestrian walkways in a small town and featured shoppers, shops and cars. The Green video featured three 5 min scenes of fields with sheep, hedgerows and a small wood. The Blue video featured three 5 min clips from a headland overlooking a beach and of views from beach height across rocks and the sea. 

### 2.4. Measures

Systolic (SBP) and Diastolic (DBP) blood pressure were measured using a Boso medicus automatic digital blood pressure monitor and recorded on paper. Following Pretty *et al.* [10], Mean Arterial Blood Pressure (MABP) was calculated using the formula MABP = DBP + [0.333 × (SBP − DBP)]. Heart rate (HR) was monitored using a Polar fitness heart rate chest strap worn throughout the trial with HR sent to a watch worn by the experimenter. 

Affective responses were measured using two scales, one for the dimension of valence (*i.e.*, positive-negative) and one for the dimension of arousal (*i.e.*, high-low; 22). Valence was measured using the single-item *Feelings Scale* (*FS*), which asks how participants are feeling “right now” on an 11 point scale ranging from “+5 = Very good” to “−5 = Very bad” [46]. Arousal was measured using the single item *Felt Arousal Scale* (*FAS*) which also asks participants how they are feeling “right now” but on a 6 point Likert scale ranging from “1 = Low arousal” to “6 = High arousal” [47]. Single item scales allowed minimal intrusion during the cycling experience. Following Vohs and Schmeichel [48], *time perception* (*TP*) was measured by asking participants how long they felt they had been cycling for in minutes and seconds. Perceived exertion was measured using Borg’s [28] *Rate of Perceived Exertion* (*RPE*) scale where 6 reflects a workload that is extremely light, whilst 20 reflects maximal effort. 

At the end of each session participants were asked to which extent they agreed with the following three statements (from “0 = Not at all” to “6 = Very much”): (a) “I enjoyed the cycle”, (b) “I feel better after doing this cycle” and (c) “I would be happy to do this cycle again”. The first and third items were adapted from Focht [20]. The three were highly correlated (Cronbach’s αs across conditions ranged from 0.81–0.89) allowing construction of a global “evaluation” of the experience scale. After completing all trials, participants were asked which of the four they would be most willing to repeat. 

### 2.5. Procedure

After being welcomed into the laboratory, the HR monitor chest strap was fitted before participants sat alone quietly for 5 min with their legs outstretched to stabilize physiological indices. Pre-exercise baseline measures of HR, SBP, DBP, FS and FAS were taken. Participants then mounted the bike and as soon as they reached their predetermined RPM the trial started. 

Apart from the Control trial, where participants merely faced the blank wall, the relevant video was started, and participants in all conditions continued to cycle at moderate intensity for 15 min. Whilst cycling, measures of HR, TP, FS, FAS and RPE (in that order) were taken at 5, 10 and 15 min (just before the videos were switched off). RPM and HR was constantly monitored to ensure participants maintained a light to moderate level of exercise intensity. A gentle instruction to “please speed up (slow down) a little” was usually needed once or twice per session. After 15 min, participants returned to the chair to sit quietly alone for 5 min. Then Post-exercise measures of HR, SBP, DBP, FA and FAS were taken and the evaluation questions asked.

Which trial participants would most like to repeat was asked at the end of the final session when they were also thanked and offered a book of local walks. Travel expenses and a summary of results were sent to all participants, but there were no further incentives. Most of the 185 sessions (5 × 37) were conducted by the third author with additional testing by the first author and two additional experimenters. 

### 2.6. Statistical Analysis

We employed omnibus, fully repeated measures Analyses of Variance (ANOVAs) for examining the effect of Time of measurement (e.g., Pre/5 min/10 min/15 min/Post) and Environment type (*i.e.*, Control/Urban/Green/Blue) on each dependent variable. We ran two types of planned contrasts to test specific effects. Simple contrasts for Time compared the outcomes for two adjacent times (*i.e.*, Pre *vs.* 5 min; 5 min *vs.* 10 min; 10 min *vs.* 15 min; 15 min *vs.* Post). This enabled us to explore how outcomes changed progressively over time. Contrasts for Environment were then based on comparisons of each of the three experimental conditions with the Control condition, to examine interactive effects of PA in different simulated “outdoor” environments compared to an “indoor” control environment. We did not directly compare the “outdoor” environments with each other as this would result in a substantial increase in the overall number of comparisons and increase the chance of Type II errors. Although this problem could be mitigated by adjusting the level of statistical significance, the adjustment needed for so many comparisons could easily result in Type 1 errors and, more theoretically, our main interest was in seeing whether exercise in specific settings was more beneficial than just exercise alone (*i.e.*, Control) than comparing exercise across different “outdoor” settings. 

## 3. Results 

Means (M) and standard deviations (SDs) for physiological variables are presented in Table 1 and those for psychological variables in Table 2. Due to the complex nature of the results, ANOVA main effects, interactions and planned contrasts, including F values, degrees of freedom and effect sizes (partial eta squares), are presented in Table 3 and Table 4. Where means are reported in the text they refer to changes in scores over time, results from conditions combined, or other data not presented in Table 1 or Table 2.

**Table 1:** Means (M) and standard deviations (SD) for physiological variables at all times points in all four conditions.

	Control (Blank Wall)		Urban Video (Town)		Green Video (Countryside)		Blue Video (Coastal)	
	M	SD	M	SD	M	SD	M	SD
MABP ^a^								
*Pre*	97.79	12.89	97.59	11.28	98.02	11.86	100.64	13.56
*Post*	98.04	10.54	94.89	11.82	95.62	10.81	96.91	12.53
SBP ^b^								
*Pre*	125.00	17.85	127.92	16.76	127.08	18.11	126.95	18.22
*Post*	124.84	15.84	121.38	15.85	121.62	17.31	122.03	17.57
DBP ^c^								
*Pre*	84.19	11.68	82.43	9.49	83.49	10.01	87.49	12.51
*Post*	84.65	9.76	81.65	10.68	82.62	8.72	84.35	10.97
Heart rate								
*Pre*	77.24	12.35	75.24	11.24	77.43	13.14	77.49	12.44
*@5 min*	101.81	12.76	97.57	12.49	100.11	13.94	101.16	13.61
*@10 min*	105.73	13.98	100.41	13.29	103.59	14.75	105.43	13.33
*@15 min*	108.31	14.36	103.38	14.69	107.49	16.07	107.86	14.85
*Post + 5 min*	80.11	11.49	79.08	9.13	80.78	11.84	79.16	11.98

^a^ MABP = Mean Arterial Blood Pressure; ^b^ SBP = Systolic blood Pressure; ^c^ DBP = Diastolic Blood Pressure.

**Table 2:** Means (M) and standard deviations (SD) for psychological variables at all times points in all four conditions.

	Control (Blank Wall)		Urban Video (Town)		Green Video (Countryside)		Blue Video (Coastal)	
	M	SD	M	SD	M	SD	M	SD
Feelings Scale								
*Pre*	3.27	1.46	2.38	1.74	2.61	1.85	2.43	1.83
*@5 min*	2.58	1.75	2.24	1.42	3.24	1.19	3.16	1.39
*@10 min*	2.68	1.56	2.14	1.60	3.32	1.16	3.30	1.23
*@15 min*	2.75	1.56	2.11	1.65	3.36	1.46	3.28	1.55
*Post + 5 min*	3.64	1.18	3.20	1.20	3.78	0.92	3.57	1.17
Felt Arousal								
*Pre*	2.84	1.07	2.95	1.00	3.05	1.08	2.97	1.09
*@5 min*	2.50	0.93	2.62	0.86	2.68	1.06	2.65	0.95
*@10 min*	2.32	0.92	2.49	1.15	2.57	1.21	2.62	1.09
*@15 min*	2.42	1.16	2.47	1.00	2.53	1.30	2.86	1.36
*Post + 5 min*	2.51	1.17	2.46	0.96	2.54	0.99	2.42	1.28
Time Perception								
*@5 min*	5.42	1.73	5.61	2.38	5.61	2.93	4.84	1.63
*@10 min*	10.95	2.32	10.84	4.11	10.97	3.44	9.68	2.62
*@15 min*	17.22	5.39	15.81	5.79	16.81	5.30	14.76	4.28
*Mean during*	*11.12*	*2.85*	*10.75*	*3.84*	*11.05*	*3.47*	*9.76*	*2.49*
RPE ^a^								
*@5 min*	11.62	1.48	11.70	1.66	11.57	1.59	11.68	1.62
*@10 min*	11.92	1.67	12.14	1.84	12.22	1.97	11.96	1.38
*@15 min*	12.28	1.70	12.27	2.08	12.42	1.63	12.49	1.59
Evaluation								
*Enjoyed*	3.18	1.44	3.41	1.67	4.49	1.37	4.95	1.05
*Feel better*	3.49	1.59	3.78	1.70	4.62	1.42	4.59	1.40
*Repeat*	3.76	1.40	3.86	1.80	4.81	1.43	5.30	0.77
*(Combined)*	*3.47*	*1.34*	*3.68*	*1.54*	*4.64*	*1.25*	*4.95*	*0.94*

^a^ RPE = Rate of Perceived Exertion.

### 3.1. Mean Arterial Blood Pressure (MABP)

MABP was analysed using a 2 (Time: Pre/post) by 4 (Environment: Control/Urban/Green/Blue) fully repeated measures ANOVAs with planned contrasts as detailed above. Consistent with previous research, the effect of Time was significant (*p* < 0.001). MABP dropped from a pre-activity level of *M* = 98.51 to a post-activity level of *M* = 96.37. Although the main effect of simulated Environment was not significant (*p* = 0.21), the interaction was marginally significant (*p* = 0.077). Supporting part of H1, the planned contrasts revealed that the change in MABP for the Blue condition (*M*_diff_ = −3.73) was significantly different (*p* = 0.014) from the change in the Control condition (*M*_diff_ = 0.25). We remain cautious however, since both Green and Urban conditions also showed marginally significant reductions in MABP compared to Control (Green *M*_diff_ = −2.40, *p* = 0.068; Urban *M*_diff_ = −2.60, *p* = 0.087). The separate effects for SBP and DBP can be seen in Table 1 and Table 3. These suggest that the benefits of Blue are due to the largest drop in DBP but that this may be due to a regression to the mean effect, as pre activity DBP was highest in this group. 

**Table 3:** Results of ANOVAs with planned contrasts for physiological variables: Repeated for Time, Simple for Environment (with Control as comparator), and Repeated × Simple for Time by Environment.

	Overall				Urban *vs.* Control				Green *vs.* Control				Blue *vs.* Control			
	(df)	F	P	_p_η^2^	(df)	F	P	_p_η^2^	(df)	F	P	_p_η^2^	(df)	F	P	_p_η^2^
MABP																
Time	(1,36)	12.59	0.001	0.26	-	-	-		-	-	-		-	-	-	
Environment	(3,108)	1.53	0.210	0.04	(1,36)	1.84	0.184	0.05	(1,36)	0.83	0.370	0.02	(1,36)	0.34	0.562	0.01
T × E	(3,108)	2.34	0.077	0.06	(1,36)	3.44	0.072	0.09	(1,36)	3.55	0.068	0.09	(1,36)	6.70	0.014	0.16
SBP																
Time	(1,36)	20.95	<0.001	0.37	-	-	-		-	-	-		-	-	-	
Environment	(3,108)	0.04	0.991	0.00	(1,36)	0.02	0.882	0.00	(1,36)	0.10	0.758	0.00	(1,36)	0.05	0.826	0.00
T × E	(3,108)	2.29	0.083	0.06	(1,36)	5.54	0.024	0.13	(1,36)	4.90	0.033	0.12	(1,36)	3.28	0.079	0.08
DBP																
Time	(1,36)	2.78	0.104	0.07	-	-	-		-	-	-		-	-	-	
Environment	(3,108)	3.98	0.010	0.10	(1,36)	4.81	0.035	0.12	(1,36)	1.54	0.223	0.04	(1,36)	1.10	0.302	0.03
T × E	(3,108)	1.73	0.166	0.05	(1,36)	0.70	0.408	0.02	(1,36)	0.74	0.394	0.02	(1,36)	4.72	0.036	0.12
Heart rate																
Time	(4,136)	289.33	<0.001	0.90	-	-	-		-	-	-		-	-	-	
Pre–5 min	(1,34)	281.95	<0.001	0.90	-	-	-		-	-	-		-	-	-	
5–10 min	(1,34)	85.89	<0.001	0.72	-	-	-		-	-	-		-	-	-	
10–15 min	(1,34)	42.00	<0.001	0.55	-	-	-		-	-	-		-	-	-	
15 min–Post	(1,34)	384.34	<0.001	0.92	-	-	-		-	-	-		-	-	-	
Environment	(3,102)	3.98	0.010	0.06	(1,34)	4.60	0.039	0.12	(1,34)	0.22	0.643	0.01	(1,34)	0.31	0.581	0.01
T × E	(12,408)	2.28	0.084		-	-	-		-	-	-		-	-	-	
Pre–5 min × E	-	-	-		(1,34)	4.29	0.046	0.11	(1,34)	2.05	0.161	0.06	(1,34)	0.56	0.458	0.02
5–10 min × E	-	-	-		(1,34)	2.61	0.115	0.07	(1,34)	0.32	0.573	0.01	(1,34)	0.24	0.627	0.01
10–15 min × E	-	-	-		(1,34)	1.21	0.282	0.03	(1,34)	2.17	0.150	0.06	(1,34)	0.04	0.838	0.00
15 min–Post × E	-	-	-		(1,34)	7.30	0.011	0.18	(1,34)	1.74	0.196	0.05	(1,34)	0.16	0.695	0.01

**Table 4:** Results of ANOVAs with planned contrasts for psychological variables: Repeated for Time, Simple for Environment (with Control as comparator), and Repeated × Simple for Time by Environment.

	Overall				Urban *vs.* Control				Green *vs.* Control				Blue *vs.* Control			
	(df)	F	P	_p_η^2^	(df)	F	P	_p_η^2^	(df)	F	P	_p_η^2^	(df)	F	P	_p_η^2^
Feelings scale																
Time	(4,140)	13.82	<0.001	0.28	-	-	-		-	-	-		-	-	-	
Pre–5 min	(1,35)	1.62	0.211	0.04	-	-	-		-	-	-		-	-	-	
5–10 min	(1,35)	0.09	0.763	0.00	-	-	-		-	-	-		-	-	-	
10–15 min	(1,35)	0.04	0.852	0.00	-	-	-		-	-	-		-	-	-	
15 min–Post	(1,35)	33.80	<0.001	0.49	-	-	-		-	-	-		-	-	-	
Environment	(3,105)	5.18	0.002	0.13	(1,34)	6.65	0.014	0.16	(1,35)	1.38	0.248	0.04	(1,35)	0.26	0.613	0.01
T × E	(12,420)	4.35	<0.001	0.11	-	-	-		-	-	-		-	-	-	
Pre–5 min × E	-	-	-		(1,35)	4.39	0.043	0.11	(1,35)	16.47	<0.001	0.32	(1,35)	15.46	<0.001	0.31
5–10 min × E	-	-	-		(1,35)	0.18	0.674	0.01	(1,35)	0.03	0.876	0.00	(1,35)	0.02	0.888	0.00
10–15 min × E	-	-	-		(1,35)	0.42	0.521	0.01	(1,35)	0.07	0.793	0.00	(1,35)	0.20	0.660	0.01
15 min–Post × E	-	-	-		(1,35)	0.84	0.365	0.02	(1,35)	2.74	0.107	0.07	(1,35)	5.81	0.021	0.14
Felt arousal																
Time	(4,140)	5.78	<0.001	0.14	-	-	-		-	-	-		-	-	-	
Pre–5 min	(1,35)	9.18	0.005	0.21	-	-	-		-	-	-		-	-	-	
5–10 min	(1,35)	1.92	0.175	0.05	-	-	-		-	-	-		-	-	-	
10–15 min	(1,35)	0.72	0.402	0.02	-	-	-		-	-	-		-	-	-	
15 min–Post	(1,35)	0.60	0.445	0.02	-	-	-		-	-	-		-	-	-	
Environment	(3,105)	0.62	0.604	0.02	(1,34)	0.38	0.541	0.01	(1,35)	1.13	0.295	0.03	(1,35)	1.93	0.175	0.05
T × E	(12,420)	0.81	0.638	0.02	-	-	-		-	-	-		-	-	-	
Pre–5 min × E	-	-	-		(1,35)	0.05	0.823	0.00	(1,35)	0.05	0.831	0.00	(1,35)	0.01	0.956	0.00
5–10 min × E	-	-	-		(1,35)	0.01	0.936	0.00	(1,35)	0.21	0.650	0.01	(1,35)	0.83	0.368	0.02
10–15 min × E	-	-	-		(1,35)	0.44	0.510	0.01	(1,35)	0.48	0.492	0.01	(1,35)	1.04	0.314	0.03
15 min-Post × E	-	-	-		(1,35)	0.06	0.814	0.00	(1,35)	0.14	0.715	0.00	(1,35)	4.76	0.036	0.12
Perceived time																
Time	(2,70)	428.16	<0.001	0.92	-	-	-		-	-	-		-	-	-	
5–10 min	(1,35)	885.97	<0.001	0.96	-	-	-		-	-	-		-	-	-	
10–15 min	(1,35)	210.59	<0.001	0.86	-	-	-		-	-	-		-	-	-	
Environment	(3,105)	2.73	0.048	0.07	(1,35)	0.41	0.528	0.01	(1,35)	0.01	0.911	0.00	(1,35)	9.37	0.004	0.21
T × E	(6,120)	1.55	0.162	0.04	-	-	-		-	-	-		-	-	-	
5–10 min × E	-	-	-		(1,35)	0.18	0.677	0.01	(1,35)	0.04	0.848	0.00	(1,35)	3.40	0.074	0.09
10–15 min × E	-	-	-		(1,35)	3.50	0.070	0.09	(1,35)	0.41	0.526	0.01	(1,35)	3.65	0.064	0.10
PRE																
Time	(2,70)	23.81	<0.001	0.41	-	-	-		-	-	-		-	-	-	
5–10 min	(1,35)	13.99	0.001	0.29	-	-	-		-	-	-		-	-	-	
10–15 min	(1,35)	23.99	<0.001	0.41	-	-	-		-	-	-		-	-	-	
Environment	(3,105)	0.09	0.964	0.00	(1,35)	0.08	0.076	0.00	(1,35)	0.35	0.556	0.01	(1,35)	0.09	0.763	0.00
T × E	(6,210)	0.75	0.609	0.02	-	-	-		-	-	-		-	-	-	
5–10 min × E	-	-	-		(1,35)	0.31	0.581	0.01	(1,35)	1.54	0.222	0.04	(1,35)	0.18	0.673	0.01
10–15 min × E	-	-	-		(1,35)	0.48	0.492	0.01	(1,35)	0.57	0.454	0.02	(1,35)	0.95	0.337	0.03
Evaluation																
Enjoyed	(3,108)	17.05	<0.001	0.32	(1,36)	0.56	0.457	0.02	(1,36)	19.12	<0.001	0.35	(1,36)	46.75	<0.001	0.57
Feel better	(3,108)	6.52	<0.001	0.15	(1,36)	0.01	0.920	0.00	(1,36)	8.72	0.006	0.20	(1,36)	7.36	0.010	0.17
Repeat	(3,108)	15.07	<0.001	0.30	(1,36)	1.23	0.274	0.03	(1,36)	17.96	<0.001	0.33	(1,36)	43.82	<0.001	0.55
(Combined)	(3,108)	16.24	<0.001	0.31	(1,36)	0.66	0.423	0.02	(1,36)	19.36	<0.001	0.35	(1,36)	35.40	<.001	0.50

### 3.2. Heart Rate

Heart rate results were analysed using a 5 (Time: Pre/5 min/10 min/15 min/Post) by 4 (Environment: Control/Urban/Green/Blue) fully repeated measures ANOVA with planned contrasts. There was a significant effect of Time (*p* < 0.001). HR rose from baseline (*M* = 76.66) to 5 min (*M* = 100.16, *p* < 0.001), from 5 to 10 min (*M* = 104.04, *p* < 0.001) and from 10 to 15 min (*M* = 106.84, *p* < 0.001), before dropping from 15 min to post exercise (*M* = 79.64, *p* < 0.001). There was a significant effect of simulated Environment (*p* = 0.01). Results of the planned contrasts suggested that this was due, in part, to a lower overall average HR in the Urban (*M* = 91.15) than Control (*M* = 94.62, *p* = 0.039) condition. There were no significant differences between Control and Green or Blue conditions. This finding was further qualified by a marginally significant Time by Environment interaction (*p* = 0.084). Again, the only significant interactions occurred between the Control and Urban conditions. In particular HR in the Urban condition increased less than in the Control condition from Baseline to 5 min (Control: *M*_diff_ = 24.57; Urban: *M*_diff_ = 22.32, *p* = 0.046) and, because it remained similarly lower across all time periods, fell less from 15 min to post (Control: *M*_diff_ = −28.40; Urban: *M*_diff_ = −24.66, *p* = 0.011). This may reflect a lack of motivation to exercise at a higher level in the Urban condition.

### 3.3. Affective Responses 

**Figure 2:**
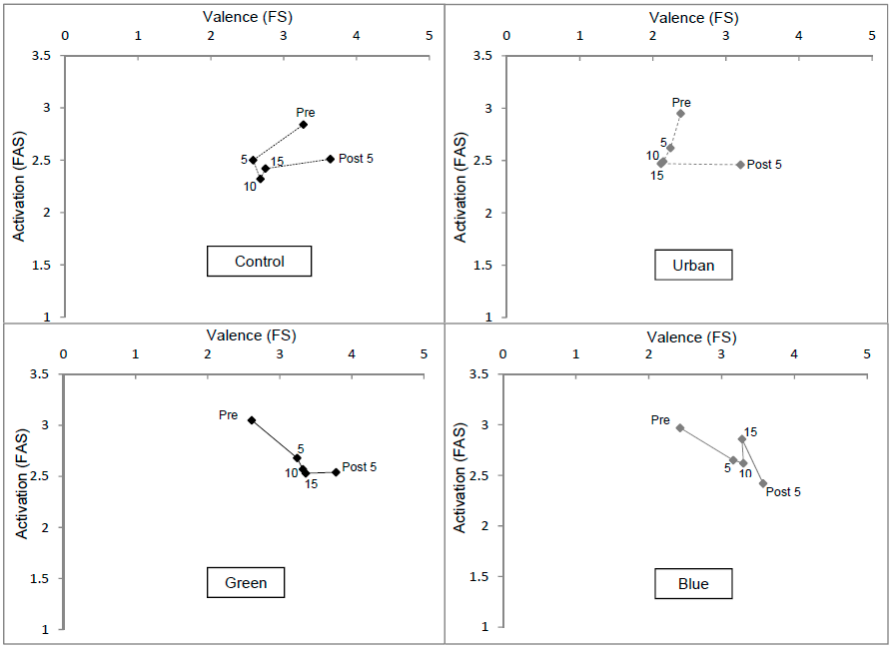
Valence (FS) and Arousal (FAS) during the four trials (times in minutes).

Affect across the four conditions, in terms of both valence (FS) and arousal/activation (FAS), is plotted in Figure 2. Broadly speaking affect became *less positive* and *less aroused* in the Control and Urban conditions during exercise. This combination is generally associated with boredom. Valence, but not arousal, then became more positive post exercise. Green and Blue simulated environments, by contrast, led to an increase in positive valence and a decrease in arousal during exercise, with a small further increase in positivity post exercise. Once established after 5 min, there were few changes in affect during the remaining 10 min of exercise. 

These patterns were analysed using two 5 (Time: Pre/5 min/10 min/15 min/Post) by 4 (Condition: Control/Urban/Green/Blue) fully repeated measures ANOVAs, one for FS and one for FAS scores. For FS there was a significant overall effect of Time (*p* < 0.001). Planned contrasts suggested the only difference between two adjacent time points was between 15 min (*M* = 2.86) and post exercise (*M* = 3.55; *p* = 0.002; all other *p*s > 0.21). This finding is consistent with earlier work finding positive increases in positive affect following exercise. There was also a main effect of simulated Environment (*p* < 0.001). Contrary to H2, however, neither the Green (*M* = 3.28, *p* = 0.248) nor Blue (*M* = 3.11 *p* = 0.613) environments resulted in significantly different patterns from Controls. Nevertheless, the Urban environment (*M* = 2.39) was associated with less positive affect than the Control environment (*M* = 2.98, *p* = 0.014), consistent with H5.

The Time by Environment interaction was also significant (*p* < 0.001). The planned contrasts revealed four significant effects. First, there were significant Pre-5 min differences between changes in feelings between the Control condition (*M*_diff_ = −0.69) and all other conditions: Urban (*M*_diff_ = −0.14, *p* = 0.043); Green (*M*_diff_ = 0.64, *p* < 0.001); Blue (*M*_diff_ = 0.73, *p* < 0.001). These findings strongly support H2 which suggested more positive affect in the Green and Blue than Control conditions. Further, that affect in the Urban condition was significantly more negative than the Control condition supports the contention that the positive effects of viewing nature during exercise are not simply due to distraction (H5). The only other significant effect was a steeper increase from 15 min to post exercise in positive affect in the Control (*M*_diff_ = 0.89) than Blue (*M*_diff_ = 0.29) condition (*p* = 0.021) which was primarily due to the already elevated level of positive affect in the Blue condition at 15 min (*M* = 3.28) compared to Control (*M* = 2.75).

There was also an overall effect of time for arousal as measured by the FAS (*p* < 0.001). The repeated contrasts revealed this was mainly due to a drop in arousal during the first five minutes of physical activity (*p* = 0.005) with no further significant decreases over time. This time there was no significant effect of Environment or a significant interaction of Environment with Time. Nonetheless, the planned contrasts did reveal a difference between Blue and Control conditions (*p* = 0.036) in the change in FAS between 15 min and Post-activity. Specifically, although there was a slight increase in arousal following exercise in the Control condition (*M*_diff_ = 0.09), the increase in the Blue condition was more substantial (*M*_diff_ = 0.44). This is consistent with the notion that the simulated Blue environment had induced a greater sense of calm and that psychologically “emerging” from that environment “back into the lab” may have undermined this state. Consequently, there does seem to be some support for H2. None of the contrasts between Control and Green or Urban were, however, significant. 

### 3.4. Time Perception

Time perception data were analysed using a 3 (Time: 5 min/10 min/15 min) by 4 (Condition: Control/Urban/Green/Blue) fully repeated measures ANOVA. The effect of time was, unsurprisingly, highly significant (*p* < 0.001). Participants felt more time had passed after 10 than 5 (*p* < 0.001) min and after 15 than 10 min (*p* < 0.001). More importantly, there was also an effect of simulated Environment (*p* = 0.048). Partly supporting H3, the planned contrasts revealed that more time was perceived to have elapsed, on average across the three time points, in the Control (*M* = 11.12) than Blue condition (*M* = 9.76, *p* = 0.004). More specifically, between 5 and 10 min participants thought, on average, that the following amount of time (in minutes) had elapsed across conditions: Control 5.53; Urban 5.23; Green 5.36; Blue 4.84. Thus while there was no significant difference between the perceived lapse of time between 5 and 10 min for the Control, Urban and Green conditions, the perceived lapse in time was marginally significantly lower in the Blue than Urban condition (*p* = 0.074). Further the lapse of time was also marginally significantly lower in the Blue than Control condition between 10 and 15 min (*p* = 0.064), resulting in a significant overall difference across time points.

### 3.5. Perceived Level of Exertion (RPE)

RPE during exercise was explored using a 3 (Time: 5 min/10 min/15 min) by 4 (Condition: Control/Urban/Green/Blue) fully repeated measures ANOVA. Again Time was highly significant (*p* < 0.001), with RPE rising from 11.62 at 5 min to 12.07 at 10 min and 12.38 at 15 min. This is in keeping with the heart rate data but also shows how participants continued to engage in only light to moderate intensity exercise. However, there was no main effect of environment or interaction. Contrary to H3, perceived effort was not significantly different in the Control condition compared to the simulated natural conditions. 

### 3.6. Evaluations and Willingness to Repeat

Evaluations were analysed using one-way ANOVAs with four levels of simulated environment and planned contrasts. Environment was significant (*p* < 0.001). Supporting H4 both the Green (*p* < 0.001) and Blue simulated environments (*p* < 0.001) were evaluated more positively than the Control condition. There was, however, no difference between the Control and the Urban environments (*p* = 0.42). For details on each of the three items separately see Table 2 and Table 4. Of note is the much larger difference between Control and Blue (F = 43.82) than Control and Green (F = 17.96) for the willingness to repeat item, indicating that participants might be especially willing to exercise again in a simulated Blue environment. Supporting this suggestion, of those who were asked which trial they would most like to repeat, 16 said Blue, 8 said Green and 1 said Urban (due to error 8 participants were not asked). A Chi Square test showed that this difference across the four trials was significant χ^2^ = 13.52, *p* < 0.001. In summary, the simulated natural environments (Green and Blue) were preferred to the Control and simulated Urban environments and the simulated Blue environment was chosen twice as often as the simulated Green environment to be the one to repeat exercise in. 

## 4. Discussion 

### 4.1. Summary of Results

Supporting previous work, our results suggested that 15 min of low-moderate intensity physical activity, in this case conducted under controlled laboratory conditions on a fixed cycle, led to positive physiological and psychological outcomes (e.g., 29). Taking the average across all four conditions, after exercise participants’ mean arterial blood pressure fell and they felt more positive and less aroused (*i.e.*, calmer/more relaxed). However, evidence of interactive benefits being greater when exposed to videos of natural environments while exercising under laboratory conditions, was mixed.

Participants’ evaluations of exercising while watching a video of a Green or Blue environment were more positive in terms of enjoyment, feeling better and willingness to repeat than in a Control environment. Watching the Urban video resulted in very similar evaluations to Control, suggesting that the more positive evaluations given to Green and Blue exercise were not simply due to having a distraction. The similarity in affective responses in the two “natural” settings is consistent with Rogerson *et al.*’s [49] field study of runners in real green (park) and blue (beach and river) settings, with a 5 km run in either setting resulting in broadly similar reductions in stress and tension and improvements in self-esteem. However, consistent with preference work [36] the most preferred setting to conduct exercise among our participants was the simulated Blue environment. Moreover, compared to the Control condition, the Blue environment was also the only one that was associated with a greater drop in mean arterial blood pressure and a perception that less time had passed. A tentative explanation is that the Blue environment was more absorbing and more likely to induce a sense of flow. As Rogerson *et al.* [49] did not measure blood pressure or time perception, whether or not these findings extend to real settings remains to be investigated. 

Simulated environment also moderated affect, especially in the first 5 min. While participants reported a reduction in positive affect during Control and Urban exercise, there was a strong increase in positive affect during the first five minutes of activity in both Green and Blue environments. Thereafter, little change in affect occurred before a further increase in positivity in all four conditions. As post exercise affective responses were similar in all conditions, had we only examined pre and post results, we may have concluded that watching videos of different environments during exercise was not important. Further, positive mood, on average, was significantly lower over the course of the trial in the Urban than Control condition suggesting that exercising in Urban settings may actually be aversive compared to a “Gym” type alternative. As well as being consistent with Pretty *et al.* [10], this finding is also consistent with a study exploring the neural correlates of viewing natural *vs.* urban scenes using functional Magnetic Resonance Imaging (fMRI) [50]. For instance, greater activation was found in the occipital lobe (lingual gyrus, middle and inferior occipital gyri), hippocampus, parahippocampal gyrus and amygdala while viewing urban *vs.* natural scenes. Based on previous studies showing greater occipital cortex and amygdala activation in association with unpleasant compared to pleasant images [51], it was inferred that greater activation in the visual cortex while viewing built images may reflect unpleasant emotions. This information may be important for promoters of physical exercise who need to be aware of the potential (relative) negative impacts on mood of exercising in different environments, especially given that many aspects of real urban exercise were not included in our study (e.g., the danger associated with cycling on a busy road) which may have attenuated our findings. Similarly, of course, such attenuation may also have applied to our Green and Blue conditions such that the effects *in situ* may be stronger than seen here, due to the soundscapes, the feelings of wind or sun and so forth.

The moderating effect of simulated environment on physiological indices was weak, replicating the conclusions of previous reviews [11,12]. Mean arterial blood pressure dropped more following Blue than Control exercise, however baseline differences, and a regression to the mean effect, may account for this. Similar patterns were seen for Urban and Green exercise. If anything, blood pressure data appear to support the view that an “outdoors” (*i.e.*, our simulated green, blue and grey settings) were better than “indoors” (*i.e.*, a blank wall similar to a gym) hypothesis, but we remain cautious not least because none of our conditions were really conducted outdoors. The limited variance in HR (and RPE) across conditions was probably due to a limitation of our methodology. Specifically, we tried to make sure participants maintained a fairly constant level of exertion over time across all trials limiting any variance that could occur across conditions. 

In sum, our results largely support previous reports of an interactive effect between exercise and exposure to different environments reflected in affective responses but not physiological outcomes. Additionally we found that these effects generalized to postmenopausal women, and that Blue (coastal) environments, even when only watched as a video, might be particularly conducive to losing track of time. More of our participants also expressed a preference to repeat the exercise in the simulated Blue setting than any other condition. Both observations might be important for the potential uptake and duration of physical activity and extend previous work that has linked exercise experience to intentions for future exercise [20,32,52]. Importantly, our results indicate that pleasure is not just a function of the exercise *per se* but is influenced by the specific settings, even when this is merely simulated. 

### 4.2. Accounting for the Effects

The current study was largely descriptive and not designed to address theoretical issues of why different interactive effects might occur. In the literature several broad explanations exist. One proposal is that exposure to natural environments promote pro-social interactions and it is these, in addition to PA itself [53], which lead to well-being enhancements [54]. As our participants exercised in isolation in an indoors setting, this would not explain our findings. 

A second possibility is that people develop a strong sense of place with specific environments [55]. This might explain the outcomes of survey work showing positive associations with well-being and self-selected visits to local parks [56], but is unlikely to account for the current findings as all participants were exposed to all environments and we have no reason to believe that any of the environments shown in the videos were particularly important for any of our individuals. Nevertheless, we recognize that all participants lived in Cornwall, a region of England close to the sea, so further work is needed to explore the generalizability of our Blue exercise findings to other populations. 

A third possibility is that both Green and Blue natural environments were intrinsically fascinating and their viewing during exercise required less cognitive resources [57]. Consequently, the mind may have been more able to “unwind” allowing participants to feel emotionally better. Although cognitive abilities were not assessed here, support for this possibility might be provided by the fact that participants generally moved to a more relaxed affective state during exercise whilst viewing natural rather than non-natural environments. This links to Ulrich’s suggestion that natural environments promote psychological and physiological stress recovery [14]. 

However, perhaps the data best fit the recent emphasis on the role of affective responses during exercise, which has been linked to future intentions [8,9,20,32,52]. Ekkekakis and colleagues in particular stress the need to go beyond a “cognitivist” paradigm and integrate hedonic approaches in our understanding of exercise behavior. For instance, differences in time perception in the Blue relative to the Control condition may have reflected feelings of awe which is known to bring people into the present moment and influence their subjective perceptions of the passing of time [58]. Further work investigating the role of specific emotions, such as awe, rather than the general mood states explored here, could therefore provide fresh insights into the underlying processes behind some of our effects. 

### 4.3. Limitations and Future Work

As noted above, there are strengths and weaknesses of using simulated “outdoor” environments (*i.e.*, videos) rather than real ones [42,43,59]. If anything, it is likely that the effects will be muted by factors that are similar across all conditions (*i.e.*, an identical room). We might then expect to see larger effects *in situ* where factors such as the breeze and smells may play a part. We also recognize that neither our participants nor experimenters were blind to condition. The former may have derived some idea as to our hypotheses and the latter may have inadvertently influenced responses. Counterbalancing of trial order, within constraints, mitigated the first possibility and standardized wording protocols mitigated the second, but we cannot rule them out completely. Future studies could address these limitations by using between-participant designs and remote data collection. 

A further limitation is that our sample of older women may not have been representative of this demographic. Their self-reported levels of physical activity per week were higher than predicted from national data [17] and they volunteered for a study involving 5 separate visits. This may indicate a particular sub-group demographic. Although careful not to over-generalize to all postmenopausal women, the findings still suggest that the environmental setting physical activity takes place in might not just be important to young students. We also note that our findings pertain to stationary cycling. Although this is a different type of exercise from walking or jogging, the similar pattern of results suggests this was not a problem. Mackay and Neill [44] found “green” cycling to be particularly good at reducing anxiety, and real cycling is widely recommended in physical activity guidelines [2]. Interestingly, in the post-study debrief with participants, most said they would be much more likely to go for a bike ride than go jogging as a form of exercise, making the study more relevant for physical activity they might actually engage in.

Future studies might also use alternative indices of physiological outcomes such as heart-rate variability (HRV), rather than heart rate *per se*, as this would enable more precise estimates to be made of parasympathetic and sympathetic nervous activity via the power levels of the high and low frequency components. Studies of Shinrin-yoku, or “forest bathing” for instance [60,61], have shown that spending time in woodlands, compared to urban environments, was associated with greater power in the High Frequency component of HRV, which is associated with parasympathetic nervous activity, and thus greater relaxation. Other indices, such as cortisol [16,61] could also be used, but these outcomes are affected by a range of circadian and other factors so care needs to be taken. Indeed in the present study we did not, for instance, monitor our participants’ food and drink, including caffeine, consumption in the hours before the trials and future studies may want to consider these issues and the impact they might have on physiological outcomes of exercise in different environmental settings. 

Finally, although we deliberately wanted to explore these issues with a sample rarely used for research in this area, *i.e.*, post-menopausal women, other groups, especially those with specific mental or physical health issues, could be the target of future studies. For instance, people with mental health issues such as depression may report less positive affect from exercising than general population samples [62]. However, studies such as these tend not to consider the environment in which such activity took place and it may thus be especially important for these groups to engage in physical activity in attractive natural environments, such as the coast, in order to experience positive emotions during and after exercise and thus be more willing to repeat it in the future. 

### 4.4. Conclusions and Implications

Our findings have implications for both public health and environmental science. If people enjoy and are willing to repeat physical activity more in natural environments, even simulated ones, then physical activity promotion campaigns might focus more on getting people out and about in nature (e.g., the Green and Blue Gym initiatives, [63]), or even providing nature videos in indoor exercise settings. This might be particularly important for particular demographic groups who engage in relatively little physical activity at present and might receive more positive affect from moderate exercise in non-specialised contexts (e.g., a park rather than a gym). Second, our results contribute to the emerging discussion surrounding *ecosystems services*, notably for health and well-being [64,65]. Greater realisation of the indirect benefits to humans of such environments might help support their protection and maintenance. Again, this highlights the critical importance of overtly recognising the intimate interconnections between the environment, health and wellbeing in public health and environmental policymaking.
